# An RNA stem-loop functions in conjunction with an upstream open reading frame to direct preferential translation in the integrated stress response

**DOI:** 10.1016/j.jbc.2022.102864

**Published:** 2022-12-31

**Authors:** Parth H. Amin, Kenneth R. Carlson, Ronald C. Wek

**Affiliations:** Department of Biochemistry and Molecular Biology, Indiana University School of Medicine, Indianapolis, Indiana, USA

**Keywords:** integrated stress response, eIF2 phosphorylation, translational control, CDS, coding sequence, CMV, cytomegalovirus, ER, endoplasmic reticulum, HEK293, Human embryonic kidney 293 cells, ISR, integrated stress response, IRES, internal ribosome entry site, RT-qPCR, reverse transcription quantitative real time PCR, SL, stem-loop, Tg, thapsigargin, uORF, upstream open reading frame, WT, wildtype

## Abstract

In response to environmental stresses, cells invoke translational control to conserve resources and rapidly reprogram gene expression for optimal adaptation. A central mechanism for translational control involves phosphorylation of the α subunit of eIF2 (p-eIF2α), which reduces delivery of initiator tRNA to ribosomes. Because p-eIF2α is invoked by multiple protein kinases, each responding to distinct stresses, this pathway is named the integrated stress response (ISR). While p-eIF2α lowers bulk translation initiation, many stress-related mRNAs are preferentially translated. The process by which ribosomes delineate gene transcripts for preferential translation is known to involve upstream open reading frames (uORFs) embedded in the targeted mRNAs. In this study, we used polysome analyses and reporter assays to address the mechanisms directing preferential translation of human *IBTKα* in the ISR. The *IBTKα* mRNA encodes four uORFs, with only 5′-proximal uORF1 and uORF2 being translated. Of importance, the 5′-leader of *IBTKα* mRNA also contains a phylogenetically conserved stem-loop of moderate stability that is situated 11 nucleotides downstream of uORF2. The uORF2 is well translated and functions in combination with the stem-loop to effectively lower translation reinitiation at the *IBTKα* coding sequence. Upon stress-induced p-eIF2α, the uORF2/stem loop element can be bypassed to enhance *IBTKα* translation by a mechanism that may involve the modestly translated uORF1. Our study demonstrates that uORFs in conjunction with RNA secondary structures can be critical elements that serve as the "bar code" by which scanning ribosomes can delineate which mRNAs are preferentially translated in the ISR.

Translational control is a rapid and efficient process by which cells reprogram gene expression. This control process involves modulation of translation factors and regulatory elements embedded in gene transcripts, which together determine translational efficiencies genome-wide. An important mechanism in translational control features phosphorylation of the α subunit of eukaryotic initiation factor 2 (p-eIF2α). Environmental stresses induce p-eIF2α, which lowers coupling of eIF2 with GTP that is required for delivery of initiator Met-tRNA_i_^Met^ to ribosomes for recognition of start codons (1). The ensuing reduction in bulk translation initiation conserves energy and nutrients and helps cells facilitate reprogramming toward adaptive gene expression. Because multiple protein kinases direct p-eIF2α and translational control in response to diverse stresses, this signaling pathway is referred to as the integrated stress response (ISR) (2).

While the ISR lowers global translation initiation, the translation efficiency of many mRNAs is not changed or is enhanced in response to p-eIF2α. In this way, there is a gradient of translation efficiencies controlled by p-eIF2α, ranging from repressed, resistant, or preferential (1, 3, 4, 5). A central gene transcript that is preferentially translated in response to p-eIF2α is *ATF4* (*CREB2*), encoding a transcriptional activator of the ISR (1, 2, 6). Induction of *ATF4* mRNA translation in response to p-eIF2α occurs by a “delayed reinitiation” mechanism that features two upstream open reading frames (uORFs) preceding the *ATF4* coding sequence (CDS) (6, 7). The 5′-proximal uORF1 is a positive-acting element that allows translating ribosomes to be retained on the *ATF4* mRNA and resume scanning for reinitiation at a downstream coding sequence (6, 8). In the absence of stress, there is lowered amounts of p-eIF2α and abundant eIF2/GTP/Met-tRNA_i_^Met^ complex, allowing scanning ribosomes to rapidly reinitiate at the next coding sequence, uORF2. The uORF2 is a negative-acting element that is situated out-of-frame with the *ATF4* CDS and thus sharply reduces *ATF4* translation. During stress, elevated levels of p-eIF2α reduces eIF2 ternary complex and delays ribosome reinitiation, permitting a portion of the scanning ribosomes to bypass the inhibitory uORF2 and instead initiate translation at the *ATF4* CDS (6, 8). Increased amounts of ATF4 protein then directs transcription of ISR target genes involved in amino acid synthesis, uptake, and reclamation, along with induction of additional transcription factors (such as CHOP also known as GADD153/DDIT3), cellular redox status, and feedback dephosphorylation of p-eIF2α through GADD34 (PPP1R15a) (2, 7, 9, 10, 11, 12, 13). Together with increased transcription *via* ATF4, *GADD34* is subject to preferential translation during p-eIF2α by a bypass mechanism involving ribosome scanning through a single inhibitory uORF that thwarts downstream CDS translation (8, 14).

In each of the preferential translation models, uORFs serve as a critical "bar code" where scanning ribosomes delineate which mRNAs are efficiently translated during the ISR (15). The precise properties of the uORFs, including coding sequence length, efficiency of translation initiation, elongation, and termination, and their placement in the 5′-leader of the mRNAs, are viewed as critical determinants for whether an uORF allows translating ribosomes to efficiently reinitiate at downstream coding sequences (8, 16, 17). Are there additional determinants that can contribute to the bar code regulating translation efficiencies in the ISR? It has been suggested that N 6-methyladenosine in the 5′-leader of mRNAs can modulate ribosome scanning and subsequent start codon selection during certain nutrient stresses (18). Furthermore, stable RNA structures can impede ribosome loading and scanning onto mRNAs, but their contributions to ISR translation are largely unknown (17, 19).

In this study, we focus on preferential translation of the ISR target gene *IBTKα*, encoding a purported CUL3-associated protein that may promote targeted protein degradation (20, 21, 22). Previously, we reported that *IBTKα* is preferentially translated in response to p-eIF2α during endoplasmic reticulum (ER) stress (3). The 5′-leader of mammalian *IBTKα* mRNA features four uORFs, and it is suggested that one or more of these may serve as inhibitory elements in translation (3). Here we show that a critical feature of preferential translation of *IBTKα* involves bypass of uORF2, which thwarts downstream reinitiation at the *IBTKα* CDS through a phylogenetically conserved RNA secondary structure that is situated just downstream of the uORF2. We conclude that RNA secondary structures can function in conjunction with uORFs to serve as critical elements of the bar code by which scanning ribosomes delineate preferential translation of gene transcripts in the ISR.

## Results

**Figure 1:**
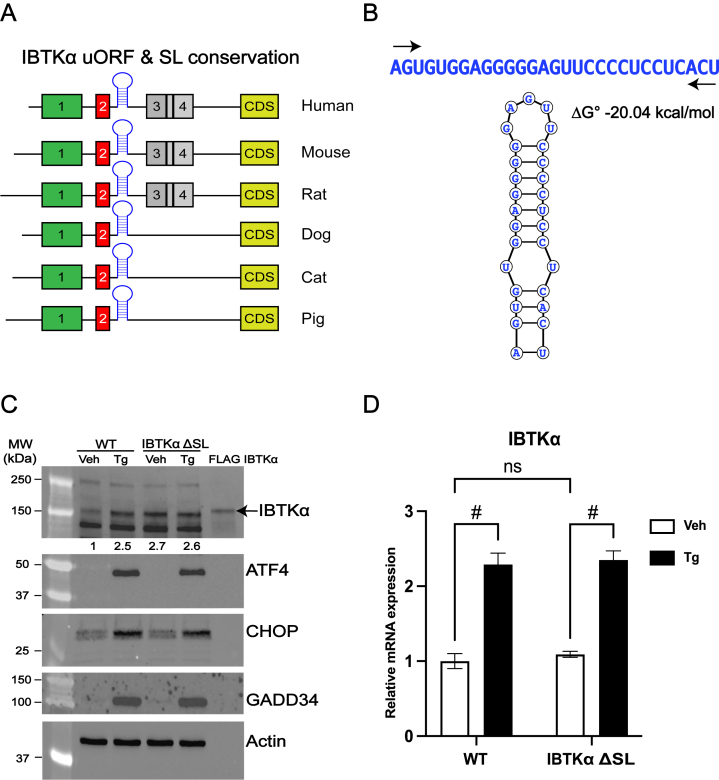

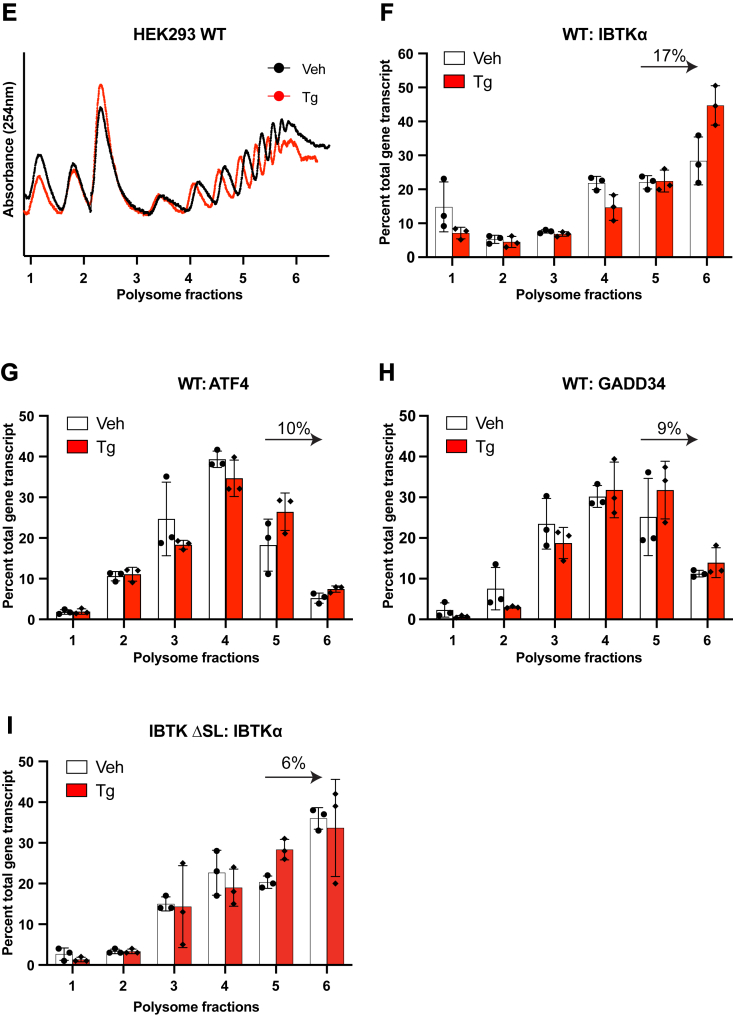
**Multiple regulatory elements are present on 5′ leader of human *IBTKα* mRNA.***A*, schematic representation of phylogenetic conservation of *IBTKα* uORFs and RNA SL among different mammalian species, *boxes* indicate the uORFs 1 to 4 and the *IBTKα* CDS for organism. The SL is indicated downstream of uORF2. The given organisms include human, mouse, rat, dog, cat, and pig. *B*, sequence of conserved SL in the *IBTKα* mRNA, along with a schematic of SL structure. The structure and the free energy values of SL were calculated using a web-based Vienna RNA secondary structure prediction software (37). *C*, WT HEK293 cells and the IBTKα ΔSL counterparts were treated with thapsigargin (Tg) or vehicle DMSO (Veh) for 8 h and the levels of IBTKα, ATF4, CHOP, GADD34, and actin proteins were measured by immunoblot analyses. Levels of IBTKα protein are shown relative to vehicle-treated WT HEK293. Lysates containing a FLAG-tagged IBTKα was used as a maker in the immunoblot assay. *D*, WT HEK293 and IBTKα ΔSL cells were treated with Tg or vehicle for 8 h, and cDNA was generated after collecting total RNA. Levels of *IBTKα* mRNA were measured by RT-qPCR and are presented as a *bar graphs* that are normalized to WT cells treated with vehicle. One-way ANOVA with Tukey’s HSD test. # Is equal to *p*-value ≤ 0.0001. WT and the IBTKα ΔSL HEK293 cells were treated with Tg or vehicle for 6 h, and cell lysates were analyzed by polysome profiling. *E*, the A_254_ profiles of the WT cells are shown, with free ribosomes, monosomes, and polysomes indicated. The average polysome to monosome ratio of three biological replicates of WT cells for vehicle or Tg-treated group is 2.8 and 1.9, respectively. *F* and *I*, RNA was isolated from the polysome fractions of WT and ΔSL cells, and the levels of *IBTKα* transcript were measured from three biological replicates of WT (*F*) and ΔSL cells (*I*) by RT-qPCR as described in the Experimental Procedures. *G* and *H*, Similarly, the levels of *ATF4* (*G*) and *GADD34* (*H*) transcripts were also measured from the WT cells. The bar graphs show the percentage of total *IBTKα*, *GADD34*, and *ATF4* mRNAs in each fraction and their shift toward heavy polysome during ER stress (*F*–*I*). The *IBTKα* mRNA shifted toward heavy polysomes (fractions 5 and 6) in response to thapsigargin treatment, with a significant increase in fraction 6 with ER stress (*p* value = 0.0074). There was trend for *ATF4* and *GADD34* transcripts toward heavy polysomes (fractions 5 and 6) with ER stress (n = 3). CDS, coding sequence; ER, endoplasmic reticulum; HEK293, human embryonic kidney 293 cells; SL, stem-loop; RT-qPCR, reverse transcription quantitative real time PCR; uORF, upstream open reading frame; WT, wildtype.

IBTKα was previously identified in a high-throughput screen for mouse gene transcripts that are preferentially translated in response to ER stress (3). A phylogenetic comparison of the 5′-leader of the *IBTKα* showed that there are four upstream uORFs present in human and rodent mRNAs, with the 5′-proximal uORF1 and uORF2 broadly conserved among mammals (Fig. 1*A*). Informatic comparisons of these *IBTK*α gene transcripts revealed that another conserved feature shared among the 5′-leaders is an RNA secondary stem-loop structure of ΔG° = −20 kcal/mol that is situated 11 nucleotides downstream of the uORF2 (Fig. 1, *A* and *B*). To address the mechanisms regulating human *IBTKα* expression during ISR and the importance of the stem-loop structure (designated SL) and the uORFs in the translational control of human *IBTKα*, we first deleted the SL in the *IBTKα* gene in human HEK293 cells using CRISPR/Cas9. The wildtype (WT) and SL-deleted cells (ΔSL) were treated with the ER stress agent thapsigargin (Tg) or vehicle, and levels of IBTKα protein were measured by immunoblot (Fig. 1*C*). Upon ER stress, WT cells showed enhanced levels of IBTKα protein that was accompanied by induced amounts of the ISR effectors ATF4, CHOP, and GADD34. By comparison, the ΔSL cells showed elevated levels of IBTKα protein independent of stress. Consistent with prior reports that *IBTKα* gene transcription is induced by ATF4 in the ISR (3), we observed similar levels of increased *IBTKα* mRNA as judged by RT-qPCR in both the WT and ΔSL cells upon ER stress (Fig. 1*D*). These results show that expression of *IBTKα* is induced by stress and ablation of the SL leads to higher amounts of IBTKα protein expression independent of the stress conditions.

We next addressed translational control of *IBTKα* using polysome profiling. We treated the WT and ΔSL cells with Tg or vehicle, and lysates were prepared and subjected to sucrose gradient centrifugation to separate well translated heavy polysomes from monosomes and free ribosomes. ER stress and accompanying p-eIF2α lowered translation initiation as viewed by a reduction in heavy polysomes (Fig. 1*E*). Measurements of human *IBTKα* mRNA in the sucrose gradient fractions from the WT cells showed a shift toward heavy polysomes during ER stress, indicative of preferential translation during bulk translation repression (Fig. 1*F*). This shift toward heavy polysomes in response to ER stress was comparable to *ATF4* and *GADD34* mRNA (Fig. 1, *G* and *H*), two targets known to be subject to translational and transcriptional induction during the ISR (1). It is noted that there was a significant shift of *IBTKα* mRNA toward the heaviest fraction 6 in response to Tg treatment (*p* = 0.0074), while there was reproducible trend that did not achieve statistical significance for *ATF4* and *GADD34* transcripts toward heavy polysomes with ER stress. In cells expressing *IBTKα* ΔSL mRNA, there was similar association of the *IBTKα* transcript with heavy polysomes independent of ER stress, with only a modest 6% shift toward the heavy polysome fractions in response to Tg treatment (Fig. 1*I*). By comparison, *ATF4* mRNA prepared from IBTKα ΔSL cells displayed a shift toward heavier polysomes in response to ER stress (Fig. S1). These results support the idea that *IBTKα* is subject to preferential translation in the ISR, and the SL embedded in the 5′-leader of *IBTKα* mRNA has a repressive function that can be at least in part overcome during ER stress.

### The uORF2 functions as a major inhibitory element in IBTKα translational control

**Figure 2:**
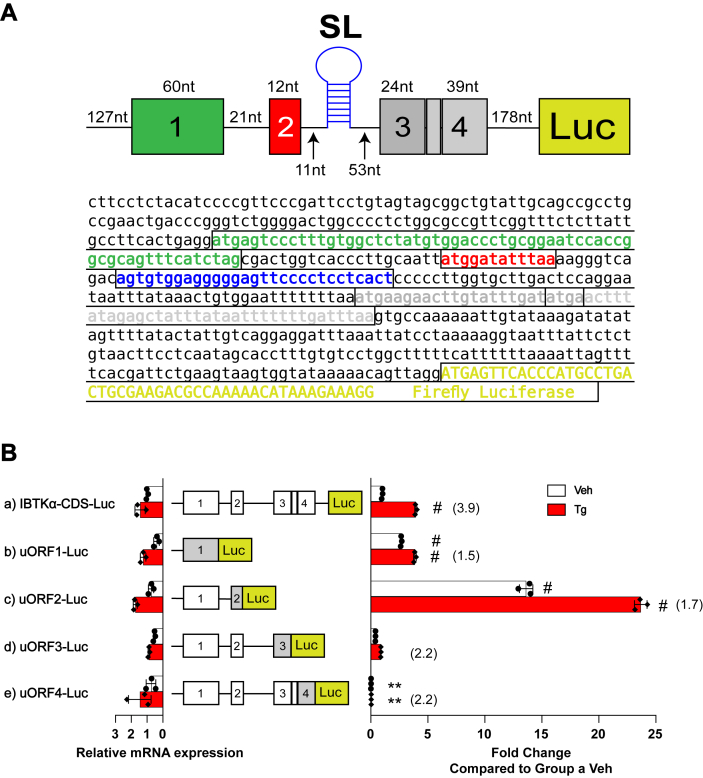
***IBTKα* mRNA is preferentially translated in response to ER stress.***A*, schematic representation of the P_CMV_-IBTKα-Luc reporter featuring the encoded 5′-leader of human *IBTKα* that was fused with the firefly luciferase CDS. The sequence of the 5′-leader IBTKα-Luc reporter is also shown with color coded uORFs and the conserved SL sequence. *B*, the P_CMV_-IBTKα-Luc reporter was co-transfected with nanoluciferase reporter into HEK293T cells and treated with Tg or vehicle DMSO for 6 h (panel a). Alternatively, the Luc CDS was fused in frame to each of the four *IBTKα* uORFs and assayed in the dual reporter system in the presence or absence of ER stress (panels b-e). Each of the reporter constructs are illustrated in the panel, and Luc expression was measured by Dual Glo luciferase in stressed (Tg, *red bars*) or vehicle (Veh, *white bars*) cells. Bar graphs are shown for each reporter, with values normalized to the IBTKα-CDS-Luc reporter expressed in vehicle-treated cells. Luciferase reporter mRNAs were also measured by RT-qPCR and are shown as bar graphs on the left side of the depicted reporter constructs. Three biological replicates are reported for each condition, with data points included in the bar graphs. The values in parentheses represent induction ratios within the same group determined as the ratio of Luc activity of Tg-treated samples compared to vehicle treated. The *symbol #* is equal to *p*-value ≤ 0.0001; ∗∗ is equal to *p*-value ≤0.01. CDS, coding sequence; ER, endoplasmic reticulum; HEK293T, human embryonic kidney 293T cells; RT-qPCR, reverse transcription quantitative real time PCR; SL, stem-loop; Tg, thapsigargin; uORF, upstream open reading frame.

To delineate the key regulatory elements directing the preferential translation of *IBTKα*, we constructed a translational reporter that contained a constitutive CMV promoter, the full-length *IBTKα* 5′-leader, and the firefly luciferase CDS (Fig. 2*A*). This reporter designated P_CMV_-IBTKα-Luc was transiently transfected into HEK293T cells in combination with a plasmid encoding nanoluciferase to aid in normalization in this dual luciferase assay. Upon Tg treatment, the IBTKα-Luc activity was increased about 4-fold, with no significant difference in the reporter mRNA as judged by RT-qPCR (Fig. 2*B*). It is noted that the nanoluciferase activity was consistently reduced by about 30% upon ER stress (Fig. S2). Nanoluciferase is a long-lived reporter, and the decrease reflects the bulk translation repression that occurs upon induced p-eIF2α during ER stress. Therefore, the reduction in nanoluciferase accounts for a small portion, albeit measurable, of the increase in the measured induction of IBTKα-Luc in response to ER stress. As expected, the addition of a small molecule inhibitor of PERK, GSK2656157 (23), lowered P_CMV_-IBTKα-Luc expression in response to Tg treatment, supporting the role of this eIF2α kinase and the ISR in facilitating its translation (Fig. S3). These results show that the IBTKα-Luc reporter can recapitulate key features of preferential translation observed during the ISR.

**Figure 3:**
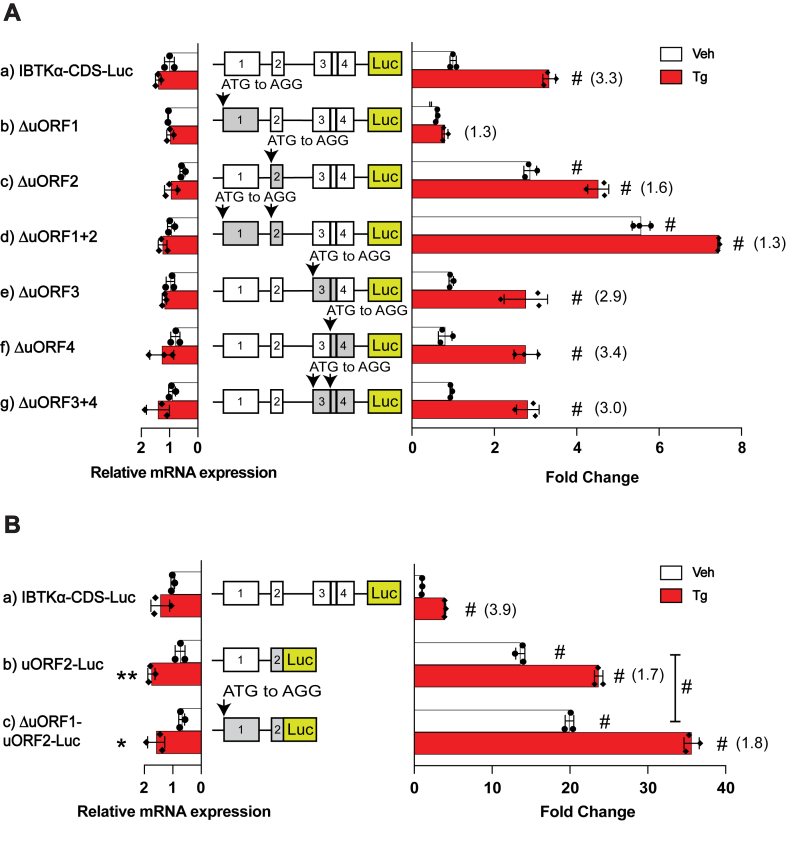
**The uORF2 functions as a major inhibitory element in *IBTKα* translational control.***A*, WT and the indicated mutant versions of P_CMV_-IBTKα-Luc reporters were assayed in HEK293T cells and treated with Tg or vehicle DMSO for 6 h. Elimination of the uORFs in the P_CMV_-IBTKα-Luc reporters was carried out individually or in combinations by substituting the initiation codons ATG to AGG. Bar graphs show luciferase expression for each reporter and values normalized to the IBTKα-CDS-Luc reporter expressed in nonstressed cells. Luc mRNAs were measured by RT-qPCR and are shown as bar graphs on the left side of the depicted reporter constructs. *B*, Luc reporters were constructed with direct fusions of uORF2 with Luc CDS in the presence of intact uORF1 (panel b) or a version with the encoded uORF1 initiation codons changed from ATG to AGG (panel c). These reporters were transfected into HEK293T cells and treated with Tg or vehicle for 6 h and bar graphs show luciferase expression for each reporter. Values are normalized to the IBTKα-CDS-Luc reporter expressed in nonstressed cells. Luc mRNAs were measured by RT-qPCR and are also shown as bar graphs. Three biological replicates are reported for each condition, with data points included in the bar graphs. Values in parentheses represent induction ratios for each reporter within the same group determined as the ratio of Luc activity of Tg-treated samples compared to vehicle treated. ∗ Is equal to *p*-value ≤ 0.05, ∗∗ is equal to *p*-value ≤ 0.01, # is equal to *p*-value ≤ 0.0001. CDS, coding sequence; ER, endoplasmic reticulum; HEK293T, human embryonic kidney 293T cells; RT-qPCR, reverse transcription quantitative real time PCR; Tg, thapsigargin; uORF, upstream open reading frame; WT, wildtype.

There are four uORFs in the human *IBTKα* mRNA, and we sought to determine whether each is translated by their direct fusion to the firefly luciferase reporter. The uORF2 is robustly translated, with more modest translation of uORF1 and minimal translation of uORFs 3 and 4 (Fig. 2*B*). These results are consistent with the phylogenetic analysis showing that uORF1 and uORF2 are conserved among mammalian *IBTKα* genes. We next addressed the roles of each uORF in the IBTKα-Luc reporter expressing the full length 5′-leader (Fig. 3*A*). To eliminate the contribution of each uORF in IBTKα translation control, the encoded initiation codon ATG was substituted to AGG. Removal of uORF1 led to an inability to induce luciferase activity in response to ER stress (Fig. 3*A* panel b). By comparison, loss of uORF2 led to robust IBTKα-Luc expression even in the absence of ER stress (Fig. 3*A* panel c). These results suggest that uORF1 can be a positive acting element in *IBTKα* translational control, whereas uORF2 is a major inhibitory element. Given that there were high levels of luciferase activity when both uORF1 and uORF2 were mutated in the reporter that were independent of ER stress, the inhibitory uORF2 function is dominant in translational control (Fig. 3*A* panel d). This result also suggests that the role of uORF1 is in part to overcome uORF2. Loss of uORF3 and uORF4 individually or in combination did not significantly alter induction of luciferase activity in response to ER stress as compared to WT (Fig. 3*A*, panels e, f, and g). These results are consistent with the minimal translation observed for uORFs 3 and 4 and indicates that these elements are not significant contributors to *IBTKα* translational control.

We next addressed whether the function of the 5′-proximal uORF1 can affect translation of the inhibitory uORF2. The uORF2 was fused to the luciferase CDS in the presence or absence of uORF1. There was high level of uORF2-Luc activity which was further elevated upon elimination of uORF1 (Fig. 3*B* panels b and c). It is noted that upon ER stress, both reporters were further enhanced. However, mRNA measurements for these reporters were also increased during the stress treatments, suggesting that increases in transcript levels were a contributor to this induction. These results suggest that part of the reason why the 5′-proximal uORF1 is a positive element is that ribosome translation of the element can in part thwart translation of the next uORF2, a major inhibitory element that is suggested to prevent reinitiation of translation at the downstream *IBTKα* CDS.

### The SL functions in conjunction with uORF2 to regulate IBTKα translation

**Figure 4:**
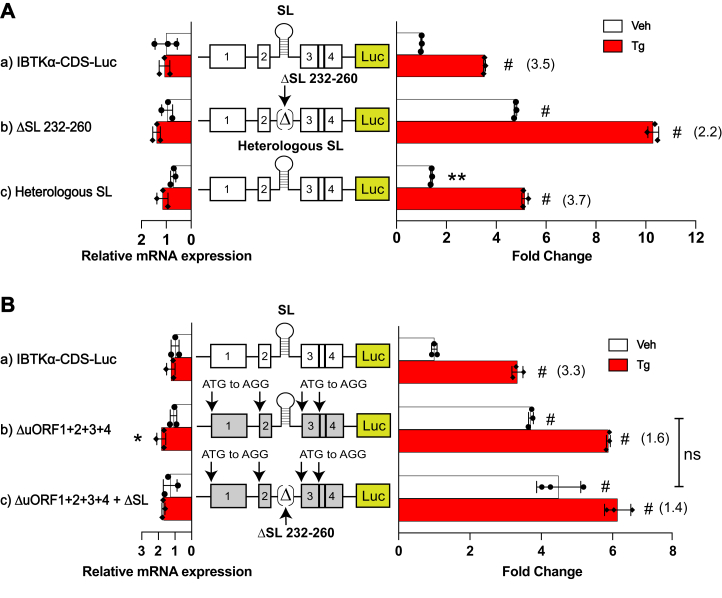
**The SL functions in conjunction with uORF2 to regulate *IBTKα* translation.***A,* mutant versions of P_CMV_-IBTKα-Luc reporters were constructed that included deletion of SL sequence (panel b, ΔSL 232–260) and a reporter in which WT SL sequence was substituted with a heterologous SL sequence with similar ΔG° (panel c). WT and mutant IBTKα-Luc reporters were transfected into HEK293T cells and treated with Tg or vehicle DMSO for 6 h. Luciferase activities are shown in bar graphs with corresponding Luc mRNA levels that were measured by RT-qPCR. Values are normalized to the IBTKα-CDS-Luc reporter expressed in nonstressed cells. *B*, mutant versions of P_CMV_-IBTKα-Luc reporters were constructed that had all four uORFs eliminated by mutation of the encoded start codons (panel b) or both loss of the uORFs in combination with deletion of the SL (panel c). Reporter plasmids were transfected into HEK293T cells and treated with Tg or vehicle for 6 h. Luciferase activities and corresponding mRNA levels are shown for each reporter. Values are normalized to the WT IBTKα-Luc expressed in nonstressed cells. There are three biological replicates depicted in the bar graphs. For each group the induction ratios are determined in response to ER stress by taking the ratios of Luc activity of Tg-treated samples compared to vehicle-treated and are indicated in parentheses. The ∗ symbol is equal to *p*-value ≤ 0.05; ∗∗ is equal to *p*-value ≤ 0.01; # is equal to *p*-value ≤ 0.0001. CDS, coding sequence; ER, endoplasmic reticulum; HEK293T, human embryonic kidney 293T cells; RT-qPCR, reverse transcription quantitative real time PCR; SL, stem-loop; Tg, thapsigargin; uORF, upstream open reading frame; WT, wildtype.

Our prior analysis of genomic deletion of the SL embedded in the 5′-leader of *IBTKα* suggested that it has a repressive function in the translational control (Fig. 1*C*). We tested this idea in the IBTKα-Luc reporter by targeted deletion of the SL element and viewed high levels of luciferase activity even in the absence of ER stress (Fig. 4*A* panel b). Inclusion of a heterologous stem-loop sequence of similar predicted stability (ΔG = -20 kcal/mol) restored to near WT levels of regulation, suggesting a SL at this position is central for the *IBTKα* translation control (Fig. 4*A*, panel c). We next addressed whether the SL by itself is a potent repressing element. Each of the four uORFs were eliminated in the IBTKα-Luc reporter, leaving only the SL element, resulting in a significant increase in expression compared to WT (Fig. 4*B*, panel a compared to b). An analogous reporter was constructed that removed the four uORFs in combination with a deletion of the SL, leading to no significant difference in IBTKα-Luc compared to the reporter eliminating only the uORFs (Fig. 4*B*, panel b compared to c). We conclude that the SL by itself is not an appreciable inhibitor of ribosome scanning and *IBTKα* translation.

**Figure 5:**
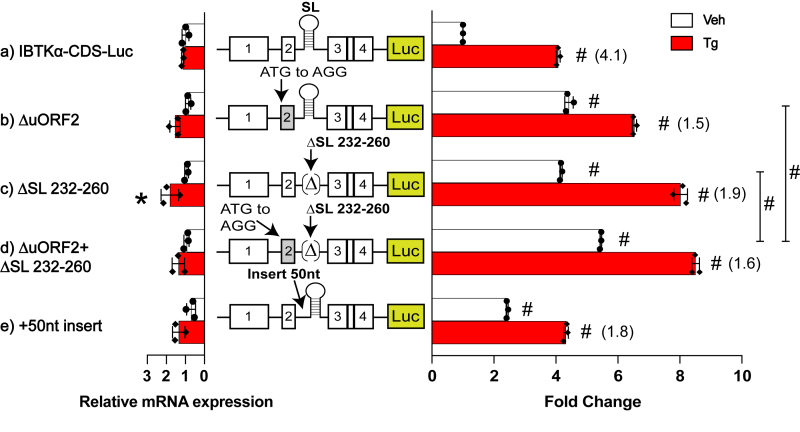
**Conserved SL regulates IBTKα translation by contributing to the repressing function of uORF2.** WT and the indicated mutant versions of P_CMV_-IBTKα-Luc reporters were transfected into HEK293T cells and treated with Tg or vehicle DMSO. Mutant versions of P_CMV_-IBTKα-Luc reporter included mutation of uORF2, a deletion of the SL sequence, and an insertion of a 50 nt segment that extended the distance between the uORF2 stop codon and the SL. Luciferase activity and corresponding Luc mRNA were measured and presented in the bar graphs that are normalized to the WT IBTKα-Luc activity in nonstressed cells. Three biological replicates are depicted in the bar graphs, with values in parentheses indicating the ER stress induction for each group calculated by taking the ratio of Luc activity of Tg treated samples compared to vehicle treated. The ∗ symbol is equal to *p*-value ≤ 0.05; # is equal to *p*-value ≤ 0.0001. ER, endoplasmic reticulum; HEK293T, human embryonic kidney 293T cells; SL, stem-loop; Tg, thapsigargin; uORF, upstream open reading frame; WT, wildtype.

We next addressed whether the SL inhibitory function is derived from its proximity to the uORF2. As noted earlier, removal of uORF2 led to high levels of IBTKα-Luc expression even in the absence of stress (Fig. 5, panel b). Deletion of the SL led to even further increase in luciferase activity (Fig. 5, panel c) that was only modestly elevated with the combined deletion of both uORF2 and the SL. Together, these observations suggest that the two elements function in combination to inhibit the downstream *IBTKα* CDS translation (Fig. 5, panel d). To address the proximity of the SL to uORF2, we adjusted the 5′-leader of the IBTKα-Luc reporter such that the SL was 50 nucleotides further downstream of the uORF2 (Fig. 5, panel e). Increasing the distance between the SL and uORF2 led to progressive higher levels of luciferase activity during the nonstressed conditions as compared to WT, whereas the induced levels of IBTKα-Luc remained similar to WT during ER stress. Insertion of 100 nucleotides between the SL and uORF2 did not further enhance luciferase activity beyond that measured for the 50-nucleotide insertion (Fig. S4). These results indicate that the proximity of the SL to the uORF2 is critical for their repressing functions in *IBTKα* translational control in basal conditions.

### The SL functions in conjunction with uORF2 to thwart translation reinitiation

**Figure 6:**
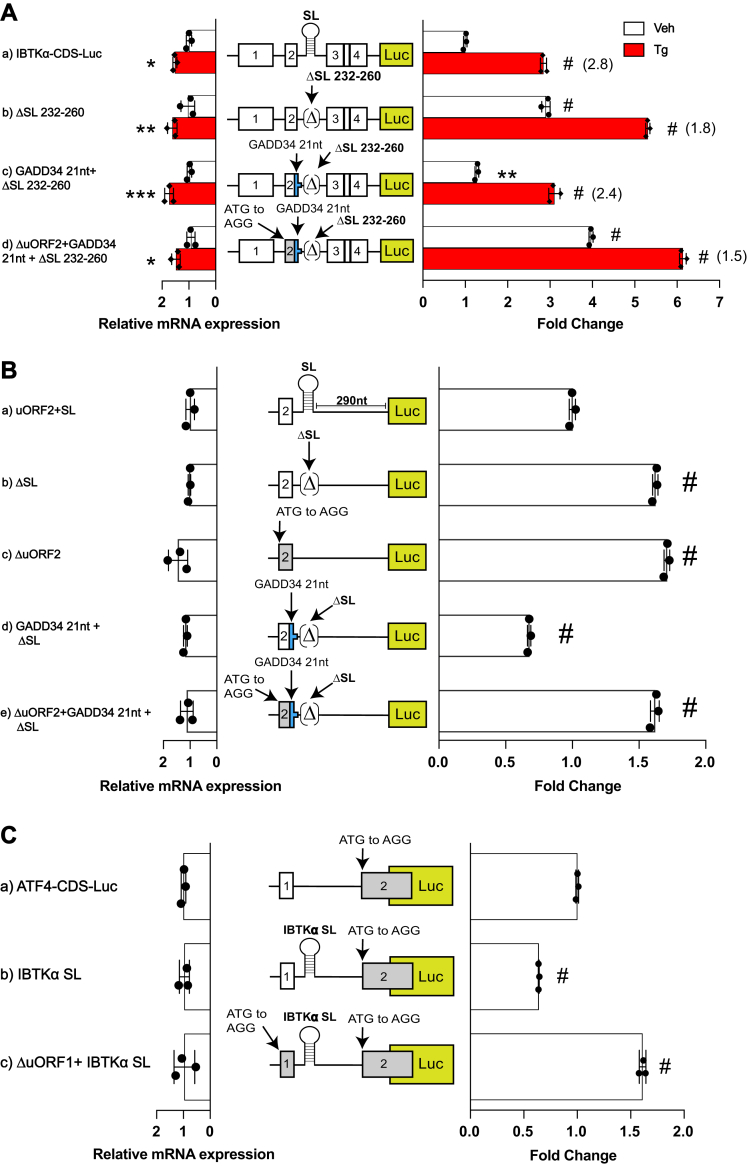
**The SL functions in conjunction with uORF2 to thwart translation reinitiation.***A*, WT and mutant versions of P_CMV_-IBTKα-Luc reporters were transfected into HEK293T subjected to ER stress for 6 h or no stress treatment. Mutant versions of the reporter that are illustrated included deletion of the SL sequence, along with substitution of the corresponding residues from the third codon of *IBTKα* uORF2 with the *GADD34* uORF2 encoding PPG∗. Furthermore, mutation of initiation codon of uORF2 was included as indicated. Luciferase activities were measured, along with levels of Luc mRNA, and are presented in the bar graphs that are normalized to the WT IBTKα-Luc activity in cells not subjected to stress. Three biological replicates are depicted in the bar graphs, and values in parentheses indicate the ER stress induction for each group calculated by taking the ratio of Luc activity of Tg treated samples compared to vehicle treated. *B*, a minimal version of the P_CMV_-IBTKα-Luc reporter featuring only the uORF2 and SL upstream of the Luc CDS was constructed as illustrated in panel a. Mutations were also prepared in the minimal IBTKα-Luc reporter that included deletion of the SL (panel b), elimination of the uORF2 (panel c), and substitution of the *GADD34* uORF2 encoding PPG∗ into the uORF2 in combination with the deletion of the SL with a functional uORF2 start codon (panel d) or with the ATG codon mutation changed to AGG (panel e). Luciferase activity and Luc mRNA activity were measured and presented in a bar that is normalized to the WT version (panel a). *C*, a P_CMV_-ATF4-Luc reporter was constructed with mutation in the uORF2 start codon to AGG, resulting in the functional loss of this inhibitory uORF (panel a). The encoded IBTKα SL was inserted 11 nucleotides downstream of the ATF4 uORF1 (panel b), and a version of this heterologous reporter was constructed with an ATG to AGG substitution in the uORF1 (panel c). This collection of ATF4-Luc reporters was transfected into HEK293T cells, and cells were cultured in the absence of stress. Luciferase activity and corresponding Luc mRNA levels were measured and are represented in a bar graph. Values are presented relative to the ATF4-Luc in panel a. The ∗ symbol is equal to *p*-value ≤0.05, ∗∗ is equal to *p*-value ≤0.01, ∗∗∗ is equal to *p*-value ≤0.001, # is equal to *p*-value ≤ 0.0001. CDS, coding sequence; ER, endoplasmic reticulum; HEK293T, human embryonic kidney 293T cells; SL, stem-loop; Tg, thapsigargin; uORF, upstream open reading frame; WT, wildtype.

Given that the SL contributes to the repressing functions of the uORF2, we posited that the SL contributes importantly to the ability of uORF2 to thwart translation reinitiation at the *IBTKα* CDS. We tested this idea using two different reporter strategies. First, we removed the SL from the IBTKα-Luc reporter and observed elevated luciferase expression even in the absence of stress (Fig. 6*A*, panel b). This idea supports the model that the SL functions to diminish ribosome reinitiation following translation of uORF2. Previously, we determined that the encoded ProProGlyStop (PPG∗) sequence in the uORF2 situated in the 5′-leader of *GADD34* is the basis for why it thwarts downstream translation reinitiation at the downstream CDS (14). In that study, we suggested that the altered termination of translation of the *GADD34* uORF2 lowered the ability of reinitiating ribosomes to be retained on the *GADD34* mRNA and resume scanning downstream for subsequent translation of the CDS (14). Of importance, the PPG∗ element is modular and can be substituted into other short uORFs to confer this repressing function (14). Following up on this idea, we substituted the PPG∗ into the uORF2 in the IBTKα-Luc devoid of the SL and determined that this substitution substantially lowered luciferase activity, consistent with lowered translation reinitiation (Fig. 6*A*, panel c). Translation of the uORF2 in this reporter was essential for this regulation because an uORF2 containing the PPG∗ but devoid of an initiation codon led to elevated levels of luciferase activity independent of stress (Fig. 6*A*, panel d).

The second reporter strategy for testing whether the SL contributes to the ability of the uORF2 to thwart reinitiation of translation involved a minimal IBTKα-Luc reporter featuring only the uORF2 and SL upstream of the Luc CDS (Fig. 6*B*). Removal of either the uORF2 or the SL led to significantly higher levels of luciferase activity compared to the WT version (Fig. 6*B*, compare panels b and c to panel a). Consistent with the earlier experiment, substitution of the PPG∗ into the uORF2 in the reporter devoid of the SL showed only low levels of luciferase expression (Fig. 6*B*, panel d). Furthermore, mutation of the initiation codon of the uORF2 containing the PPG∗ led to elevated levels (Fig. 6*B*, panel e). Together, these results suggest that either inclusion of the SL or the PPG∗ was important for uORF2 to confer impaired downstream reinitiation of translation.

Finally, we asked whether SL function is modular, similar to the ability of PPG∗ to alter translation reinitiation at an uORF. We tested this idea using the uORF1 in *ATF4*, which is a short coding sequence that is well translated and allows for efficient reinitiation of translation at downstream coding sequences (6, 7). In this reporter system, we eliminated the repressing uORF2 by mutation of the encoded initiation codon from ATG to AGG and relied on the ATF4-CDS for a measure of reinitiation (Fig. 6*C*). Substitution of the SL into the uORF1-Luc reporter significantly diminished luciferase activity (Fig. 6*C*, compare panels a and b). Removal of the uORF1 but retention of the SL sharply enhanced luciferase expression, indicating again that the SL by itself is not repressing, but rather its proximity downstream of uORF1 allows for the combined elements to thwart translation reinitiation. We conclude that the SL is modular and can lower translation initiation when placed downstream in proximity with an uORF.

## Discussion

**Figure 7:**
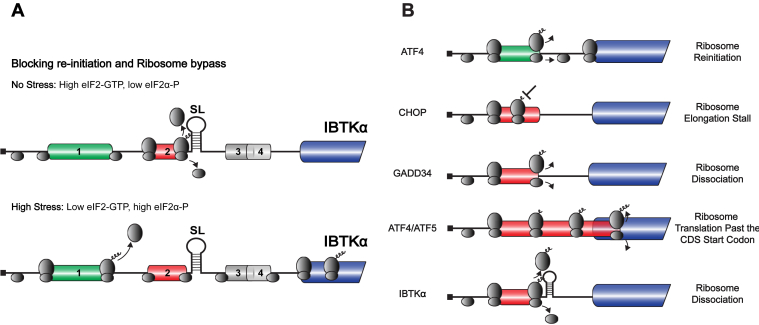
**Role of uORF2 and SL in the preferential translation of IBTKα.***A,* The uORFs in the *IBTKα* mRNA are represented as *boxes* situated upstream of the CDS encoding the IBTKα protein. In the absence of stress, there is low p-eIF2α and abundant eIF2-GTP to deliver initiator tRNA to the translational machinery. In this condition, the preinitiation ribosome complex containing 40S ribosomal subunits is thought to bind to the 5′-end cap structure, processively scan, and predominantly translate uORF2. In the illustrated model, the 40S ribosomal subunit recognizes the start codon of uORF2 (*red box*) and subsequently joins with the 60S subunit to proceed with translation elongation of the short uORF2 coding sequence. The uORF2 is a major inhibitor of *IBTKα* CDS translation and functions in conjunction with the downstream SL to sharply reduce translation reinitiation at downstream coding sequences. Therefore, upon translation of uORF2, ribosomes dissociate from the mRNA, and there is reduced translation at the downstream *IBTKα* CDS. Upon stress and enhanced p-eIF2α levels, there is reduced eIF2 ternary complex. It is suggested that some scanning ribosomes can translate the 5′proximal uORF1 (*green box*), allowing for efficient reinitiation at downstream coding sequences. Translation of uORF1 precludes translation of the nearby downstream uORF2. Once the retained scanning ribosomes proceed through the inhibitory uORF2, there is reacquisition of the eIF2/GTP/Met-tRNA_i_^Met^ complex that facilitates recognition of the start codon of the *IBTKα* CDS and enhanced synthesis of the IBTKα polypeptide. It is important to note that there is minimal translation of uORF3 and uORF4 (indicated as *gray boxes*) and their removal does not affect preferential translation of human *IBTKα*. Furthermore, while uORF3 and uORF4 are present in humans and rodents, they are absent in other mammals, supporting the idea that these elements are dispensable for *IBTKα* translational control. An additional mechanism enhancing *IBTKα* translation may involve p-eIF2α-induced ribosome bypass or leaky scanning through the uORFs and direct ribosome initiation at the *IBTKα* CDS. In this illustration, scanning and elongating ribosomes are illustrated by the ovals. *B*, uORFs in the ISR can perform different functions, including promoting ribosome reinitiation after uORF translation (*e.g.,* uORF1 in *ATF4* (6)), ribosome elongation stalling during translation of an uORF as described for *CHOP* (33), altered translation termination, and enhanced ribosome dissociation from the mRNA as reported for *GADD34* (14). Additionally, as described for the *ATF4* and *ATF5* uORF2, uORFs can be situated out-of frame with the CDS and therefore uORF translation precludes that of the CDS (6). This study shows that the *IBTKα* uORF2 combines with a downstream SL to sharply lower ribosome reinitiation at downstream coding sequences. In this illustration, the CDS is indicated by the *blue bar*. Positive-acting uORFs are indicated by a *green bar*, whereas negative-acting uORFs are denoted as *red bars*. Scanning and elongating ribosomes are illustrated by the *gray ovals*. This illustration was derived from a figure presented in a review by Young and Wek (8). CDS, coding sequence; SL, stem-loop; uORF, upstream open reading frame.

A critical element of the bar codes of mRNAs is uORFs that serve to direct the translation efficiencies in response to p-eIF2α. In this study, we addressed processes contributing to the preferential translation of human *IBTKα* mRNA during ER stress. Among four uORFs, uORF2 was shown to be well translated and serve as major inhibitory element that hinders translation reinitiation at the downstream *IBTKα* CDS (see model in Fig. 7*A*). However, the uORF2 encodes only a three-residue polypeptide and by itself does not appear to be an efficient barrier of translation. Rather uORF2 functions in conjunction with a stem-loop structure that is just 11 nucleotides downstream and together these elements are critical for thwarting downstream ribosome reinitiation. Both the uORF2 and the SL are well conserved phylogenetically, supporting the idea that they work synergistically in *IBTKα* translational control. The *IBTKα* SL is modular and can be introduced downstream of the uORF1 in the *ATF4* mRNA to change it from an uORF that allows for efficient translation reinitiation to one that prevents downstream translation. These results indicate that the bar code conferring translational control can include uORFs and RNA secondary structures, which can function in combination to modulate translation efficiencies during the ISR. Along with uORF2, *IBTKα* uORF1 is modestly translated, and it is suggested to function antagonistically to uORF2, such that translation of uORF1 precludes initiation at the nearby uORF2 (Fig. 7*A*). These processes may be a contributor to the bypass of uORF2 that is suggested to occur in response to ER stress.

### RNA secondary structures in translation regulation

Secondary structure embedded in 5′-leaders of mRNAs can influence efficiencies of translation initiation (17). While strong stem-loop structures can delay scanning of ribosomes, RNA structures of intermediate stability, such as that observed for *IBTKα* mRNA, do not alone appear to appreciably interfere with the scanning-dependent initiation process (19). There are examples whereby RNA stem-loop structures just downstream of the start codon can pause the scanning 40S ribosome, leading to enhanced time for ribosomes to recognize initiation codons in poor context (16, 17, 24, 25). Furthermore, paused ribosomes can trigger collisions with subsequent scanning ribosomes, which will then culminate in increased "dwell times" of the lagging ribosome and translation at suboptimal start codons. In the example of the uORF2/SL configuration in *IBTKα* mRNA, the context of the uORF2 start codon is optimal and the uORF2-Luc reporter devoid of the SL is well translated, which argue against the model that the SL serves to enhance scanning ribosome recognition and translation of the uORF2.

RNA structures are also instrumental for internal ribosome entry sequences (IRES) to facilitate translation initiation independent of the 5′-cap of mRNAs (26). By direct recruitment of translation initiation factors and 40S ribosomal subunits, the IRES facilitate initiation of translation by mechanisms that do not involve ribosome scanning. In the examples of picornaviruses, the IRES allow viruses to usurp the host translational machinery in favor of viral translation and replication (27). Certain cellular mRNAs also can possess IRES that may interface with the ISR. For example, the cationic amino acid transporter CAT1 mRNA was suggested to have IRES with an embedded uORF. Translation of the uORF and subsequent elongation pausing during p-eIF2α and stress is suggested to alter the IRES structure to favor translation of the *CAT1* CDS (28, 29). Therefore, in the CAT1 example, an uORF flanking RNA structures can enhance translation *via* internal ribosome loading mechanisms.

### RNA elements that confer preferential translation in the ISR

There are several properties of translated uORFs that may enhance or deter translation reinitiation. An important goal is to decipher informatic tools to predict translation efficiencies of mRNAs in the ISR based on their 5′-leader sequences. In general, short uORFs are thought to retain critical initiation factors, such as eIF3, which enhance translation reinitiation (17, 30, 31). Alternatively, as reported for the ISR regulator CHOP, short uORFs can also encode specific codons that are thought to trigger elongation pauses that lead to poor ribosome reinitiation (32, 33) (Fig. 7*B*). In the example of *GADD34*, the primary translated uORF2 encodes proline-rich sequence just prior to the stop codon. This sequence is thought to alter termination processes, hindering translation reinitiation at the downstream CDS (14). Furthermore, *ATF4* and the related *ATF5* mRNAs have an inhibitory uORF2 that overlaps out-of-frame with the CDS, which would preclude ribosome recognition of the CDS start codons (6, 34) (Fig. 7*B*). Our study adds an additional mechanism whereby an RNA stem-loop structure just downstream of an uORF can work together to impede reinitiation (Fig. 7*B*). The folding stability of the SL in the *IBTKα* mRNA is ΔG° = -20 kcal/mol, which did not preclude ribosome scanning by itself. Depending on the placement, insertion of more stable stem-loop structures can function as a strong barrier to ribosome scanning (19). Therefore, along with the position downstream of uORF2 in the 5′-leader of the *IBTKα* mRNA, folding stability is suggested to be optimized to allow for the SL to work in synergy with the translated uORF2 to serve as a negative-element in the *IBTKα* translational control. As a consequence, the model for *IBTKα* translational control presented here suggests that future mechanistic studies of the translational changes in the ISR genome-wide should integrate not only translated uORFs but also the proximity and stability of RNA secondary structures.

## Experimental procedures

### Cell culture

Wildtype human embryonic kidney cells with SV40 T antigen (designated HEK293T in this study) were purchased from ATCC (Cat No CRL-3216). Cells were grown in Dulbecco's Modified Eagle Medium media (Corning, Cat No 10–013-CV) supplemented with 10% (v/v) fetal bovine serum (Corning, Cat No 35–010-CV) and 100units/ml penicillin and 100ug/ml streptomycin (Cytiva, Cat No SV30010). Human IBTKα KO and IBTKα ΔSL (SL deleted) HEK293 cells were obtained from Synthego. The RNP-based approach was used to create an indel in exon 2 of the IBTKα gene, resulting in a frameshift in the coding sequence. In this method, guide RNAs (Table S1) targeting specific regions of human *IBTK* gene were used to in a complex with Cas9 protein. The Cas9-guide RNA complex was transfected in the WT HEK293 using a nucleofector. Mixed pools of HEK293 IBTKα KO cells, IBTKα ΔSL cells, and WT counterparts were further expanded in culture, and the knock-out efficiency were calculated by isolating genomic DNA that was used as a template to amplify indel portion by PCR using primers specific for *IBTK* edited region. The ICE software available on Synthego website (35) was used to calculate the percentage of indels in the HEK293 IBTKα KO and IBTKα ΔSL pooled cells relative to WT control cells. We created stable HEK293T cells constitutively expressing FLAG-IBTKα under the control of CMV promoter by integrating the expression cassette into the genome. Furthermore, protein lysates were prepared from control WT, IBTKα KO, IBTKα ΔSL, and FLAG-IBTKα overexpressing cells and the levels of IBTKα protein were measured by immunoblot analyses as described below.

### Immunoblot analyses

For immunoblot analyses, 2.5 × 10^6^ WT, IBTKα KO, and IBTKα ΔSL HEK293 cells were seeded in a 10 cm tissue culture dish (Corning, Cat No 353003) and cultured in complete Dulbecco's Modified Eagle Medium media. Cells were grown to about 70% confluency and then treated with 1 μM Tg (Sigma-Aldrich Cat No T9033) or vehicle DMSO (Thermo Fisher Scientific, Cat No BP-231–100). Following 8 h of treatment, cell lysates were prepared by using 120 μl of RIPA buffer solution supplemented with Halt protease and phosphatase inhibitors (Thermo Fisher Scientific, Cat No 78440) and 1 mM DTT. Cells were removed from the dish using a cell scrapper, and protein lysates were collected in 1.5 ml microfuge tubes and sonicated with 10 pulses while on ice. Lysates were then clarified by centrifugation at 12,000 rpm at 4 °C with Eppendorf centrifuge (5425 R). Protein concentrations were measured using DC protein assay kit (Bio-Rad, Cat No 5000112) according to the manufacturer’s protocol. Protein samples were prepared in Laemmli protein sample buffer (Bio-Rad, Cat No 1610747) with a final concentration of 1 μg/μl, and 20 μg of protein lysate of each were separated by electrophoresis in a SDS polyacrylamide gel. Measurements of IBTKα used Bio-Rad 4 to 15% Mini protean TGX stain-free precast protein gels (Cat No 4568084), while other proteins measured used 10% SDS polyacrylamide gels. After transferring the proteins to filters, immunoblot measurements were carried out using the following primary antibodies: IBTKα (1:1000, Thermo Fisher Scientific Cat No PA5-24224), ATF4 (1:1000, In-house (34)), CHOP (DDIT3) (1:500, Abcam Cat No ab11419), total eIF2 (1:3000, CST Cat No 5324S), p-eIF2α (1:500, Abcam Cat No ab32157), GADD34 (1:2000, Proteintech Cat No 10449-1-AP), beta-actin (1:10,000, Sigma-Aldrich A5441). The antibody validation of IBTKα was carried out by immunoblot from the lysates prepared from IBTKα ΚΟ cells treated with Tg or vehicle and IBTKα overexpressing cells (Fig. S5). Following washes in TBS solution supplemented with 0.1% tween 20, targeted proteins were visualized using the respective HRP-conjugated secondary antibody in combination with Bio-Rad Clarity Western ECL substrate (Cat No 1705060). Images were attained *via* a Bio-Rad ChemiDoc Imaging System. IBTKα immunoblots were quantified by Image J, and the relative levels of IBTKα among the samples were calculated with actin serving as a normalizing control.

### Plasmid constructions

A cDNA segment encoding the 550-nt long 5′-leader sequence of the human *IBTKα* gene transcript was inserted between the CMV promoter and the firefly luciferase CDS in pcDNA5 vector using the infusion cloning kit from Takara (Cat No 638947). The resulting P_CMV_-IBTKα-Luc reporter plasmid was transiently transfected into HEK293T cells and luciferase assays preformed as described below. Similarly, the 5′-leader of the mouse *ATF4* mRNA was inserted into the pcDNA5 vector between the CMV promoter and the firefly luciferase CDS. The WT IBTKα and ATF4 reporters were used as templates to construct the mutant versions described in the Table S2. Mutations and insertions in the 5′-leader were created using GBlock segments featuring the cDNAs encoding the 5′-leaders or *via* site directed mutagenesis using the NEB Q5 site-directed mutagenesis kit (Cat No E0554S). Each of the luciferase reporters were verified by Sanger Sequencing.

### Luciferase reporter assays

For luciferase report assays, six-well culture plates (Corning Cat No 3516) were coated with Poly-D-lysine (PDL) from Sigma-Aldrich (Cat No P6407). 700 μl of a solution of 10 μg/ml PDL was added per well, and the plate was then incubated for 20 min at room temperature. Following incubation, the PDL was aspirated out of the plates, and the wells were washed with sterile culture grade water. The plates were dried for 1.5 h by partially keeping the lid open in the culture hood. Next, 2 × 10^5^ HEK293T cells were seeded per well. The following day, WT and mutant versions of IBTKα firefly luciferase reporters were cotransfected with pNL1.1-PGK-nanoluciferase (Promega Cat No N1441) with the ratio of 1:100, respectively, using FuGENE 6 transfection reagent (Promega Cat No E2691). For each luciferase reporter, two plates of cells were transfected, one plate was used for collecting cell lysate for luciferase assay and one was used for RNA isolation for qPCR measurements of the reporter mRNA. Post 24 h of transfection (day 3), cells were treated with 0.1 μM Tg or 2 μM PERK inhibitor GSK2656157 (23) or DMSO vehicle for 6 h. Following treatment, media were removed by aspiration from the plates, and cells were washed with PBS solution. 500 μl of 1× passive lysis buffer solution (Promega Cat No E1941) was then added to each well to collect cell lysates for the luciferase assays. Plates were placed on an orbital shaker at 100 rpm for 15 min to ensure cell lysis. At least three biological replicates were carried out for each luciferase assay. Cell lysates were transferred to 1.5 ml microfuge tubes. After clarifying lysates by centrifugation, 20 μl of the supernatant was used for measuring firefly luciferase and nanoluciferase values using a luminometer. The firefly and nanoluciferase values were determined, and the firefly to nanoluciferase ratios were calculated for each. The firefly to nanoluciferase ratio of vehicle-treated WT IBTKα-Luc group was adjusted to 1 and served as the nonstressed control. The fold change for each the other groups were calculated by normalizing their firefly to nanoluciferase ratios to that of DMSO-treated WT IBTKα-Luc control group.

### Polysome profiling and sucrose gradient ultracentrifugation

2.0 × 10^6^ WT and IBTKα ΔSL HEK293 cells were seeded in 10 cm culture dish, and on the next day, cells were treated with 1 μM Tg or vehicle DMSO for 6 h. Thirty minutes prior to lysate collection, cells were treated with 50 μg/ml cycloheximide. Cells were collected and washed with cold PBS containing 50 μg/ml cycloheximide, and then 500 μl of polysome lysis solution (20 mM Tris pH 7.5, 100 mM NaCl, 10 mM MgCl_2_, 0.4% Nonidet P-40, and 50 μg/ml cycloheximide) was added per 10 cm dish to lyse the cells. Crude lysate was scrapped with cell scrapper and collected in microfuge tubes. Lysates was sheared with sterile syringe with 23-gauge needle and clarified by centrifugation at 10,000*g* for 15 min. Next, 400 μl volume of the clarified supernatant was layered onto the top of a 10 to 50% sucrose gradient, followed by ultracentrifugation at 38,000 rpm using Beckman SW41Ti rotor for 2 h at 4 °C. Fractionation of the sucrose gradient was carried out as previously described (3, 36) using a Piston Gradient Fractionator (Biocomp), and a 254-nm ultraviolet monitor with Data Quest software was used to monitor the polysome profile. A total of 12 fractions were collected from each sample, which were combined pairwise for a total of six fractions. To measure the shift in *IBTKα*, *ATF4*, and *GADD34* mRNA in the polysome fractions in response to Tg treatment, 3 μl of 1 μg/ml firefly luciferase control RNA (Promega Cat No L4561) was spiked into each pooled sample to ensure for PCR normalization between fractions. 750 μl of Trizol LS (Thermo Fisher Scientific Cat No 10296010) was added per 250 μl of pooled sample, and RNA isolation and cDNA generation was performed as mentioned detailed in the RNA isolation and RT-qPCR methods section below. The percent total gene transcript was calculated as described previously (3, 36). Briefly, the percentage shift in *IBTKα*, *ATF4*, and *GADD34* mRNA was calculated as (percentage total mRNA in fractions 5–6 during ER stress/the total mRNA levels) – (percentage total mRNA in fractions 5–6 during no stress/the total mRNA levels). It is noted that the human *IBTKα* mRNA encodes a large CDS (1353 codons), whereas *ATF4* and *GADD34* transcripts are shorter. The *ATF4* CDS contains 351 codons, while *GADD34* expresses two isoforms, the longest of which is 674 codons in length. Consequently, translated *IBTKα* transcripts are more abundant in the heavy fraction 6 of the sucrose gradients. The polysome to monosome ratio was calculated by measuring area under the curve of the polysome trace. The polysome fraction is defined as the portion of the trace that contains the trisome peak and all peaks after that.

### RNA isolation and RT-qPCR

To measure the levels of *IBTKα* mRNA, HEK293 WT and IBTKα ΔSL cells were treated with Tg or DMSO for 6 h, and total RNA was collected using Qiagen RNeasy kit (Cat No 74104) according to manufacturer’s protocol. For luciferase reporter mRNA, total RNA was collected from Tg or DMSO-treated HEK293T cells that were transfected with different IBTKα or ATF4-driven reporters using TRIzol (Thermo Fisher Scientific Cat No 15596026) according to the manufacturer’s protocol. At least three biological replicates were conducted for each measurement. The concentration of RNA for all the samples were measured by Nano drop spectrophotometer (Thermo Fisher Scientific). A total of 1 μg of RNA was used for cDNA synthesis using High-Capacity cDNA Reverse Transcription kit (Thermo Fisher Scientific, Cat# 4368813) according to manufacturer’s protocol. Following cDNA synthesis, samples were diluted five-fold with molecular biology grade water. Five microliter of total 200 μl diluted cDNA was used per well for performing qPCR. The PCR amplification was carried out by PowerUp SYBR Green master mix (Thermo Fisher Scientific, Cat# A25742) using an Applied Biosystems QuantStudio 5 Real-Time PCR system. The primers specific for the targets are mentioned in the Table S1. The relative abundance for each transcript was calculated by ΔΔCT method with *GAPDH* serving as an internal control.

### Statistical analysis

Significant differences in transcripts associated with heavy polysomes were measured by the Šídák's multiple comparisons test. Values in the luciferase reporter assays represent the mean ± standard deviation and indicate at least three independent experiments. Two-way ANOVA followed by a post hoc Tukey’s HSD test was used to analyze differences between multiple groups. *p* values less than 0.05 were considered statistically significant.

## Data availability

Data are presented within the manuscript and plasmids, and other reagents are available for academic purposes upon request.

## Supporting information

This article contains supporting information, including tables and figures in a single file.

## Conflict of interest

R. C. W. is a member of the advisory board of HiberCell.
