# Supplementary material for: An RNA stem-loop functions in conjunction with an upstream open reading frame to direct preferential translation in the integrated stress response
Source: J Biol Chem. 2022 Dec 31;299(2):102864. doi: 10.1016/j.jbc.2022.102864 (PMC9971878; doi:10.1016/j.jbc.2022.102864)
Supplement: Supplemental information [file mmc1.docx]

**SUPPORTING INFORMATION**

An RNA stem-loop functions in conjunction with an upstream open reading frame to direct preferential translation in the integrated stress response

Parth H. Amin^1^, Kenneth R. Carlson^1^, and Ronald C. Wek^1^

^1^Department of Biochemistry and Molecular Biology, Indiana University School of Medicine, Indianapolis, IN 46202, USA.

**Supplemental Table 1: List of IBTKα guide sequences and primer sequences.**

| **CRISPR guides targeting human *IBTK* gene** | | |
| --- | --- | --- |
|  | Target | Guide Sequences |
| 1 | IBTKα-Guide1 | TTTGATGTGCAGTCAGGCAT |
| 2 | IBTKα-Guide2 | CTTTGATGTGCAGTCAGGCA |
| 3 | IBTKα-Guide3 | CGACACTTTGATGTGCAGTC |
| 4 | IBTKα-Guide4 | GACATCCAAAGCATGCTTCA |
| **CRISPR guides targeting SL region of human IBTKα 5’-leader** | | |
|  | Target | Guide Sequences |
| 1 | IBTKα-SL-Guide1 | TCCTGGAGTCAAGCACCAAG |
| 2 | IBTKα-SL-Guide2 | AAAAGGGTCAGACAGTGTGG |
| **Primers for sanger sequencing of genomic DNA** | | |
|  | Target | Primer Sequences |
| 1 | IBTKα-gDNA-FP | AGGTCACCCTTGCAATTATGGATA |
| 2 | IBTKα-gDNA-RP | ATCCACTCCTTTCTGAATAAGCCA |
| **RT-qPCR primers** | | |
|  | Target | Primer Sequences |
| 1 | IBTKα-FP | CCGCCTTCCAGTTGTAATG |
| 2 | IBTKα-RP | AGCAAACAACCCAGTTGTCC |
| 3 | Firefly Luciferase-FP | CCAGGGATTTCAGTCGATGT |
| 4 | Firefly Luciferase-RP | AATCTCACGCAGGCAGTTCT |
| 5 | GAPDH-FP | AGGGCTGCTTTTAACTCTGGT |
| 6 | GAPDH-RP | CCCCACTTGATTTTGGAGGGA |

**Supplemental Table 2: List of different IBTKα-Luc and ATF4-Luc reporters**

| **Gene Construct** | **Description of mutation** |
| --- | --- |
| **Reporters in Figure 2B** | |
| IBTKα-CDS-Luc | 550nt long 5’ leader of Human IBTKα along with first 8 codons of IBTKα inserted between CMV promoter and firefly luciferase CDS |
| IBTKα-uORF1-Luc | uORF1 fused in frame to Luc CDS from the 19^th^ codon of uORF1 |
| IBTKα-uORF2-Luc | uORF2 fused in frame to Luc CDS from the 3^rd^ codon of uORF2 |
| IBTKα-uORF3-Luc | uORF3 fused in frame to Luc CDS from the 7^th^ codon of uORF3 |
| IBTKα-uORF4-Luc | uORF4 fused in frame to Luc CDS from the 12^th^ codon of uORF4 |
| **Reporters in Figure 3A-derived from IBTKα-CDS-Luc** | |
| IBTKα-ΔuORF1 | Initiation codon ATG to AGG of uORF1 |
| IBTKα-ΔuORF2 | Initiation codon ATG to AGG of uORF2 |
| IBTKα-ΔuORF1+2 | Initiation codon ATG to AGG of uORF1 and uORF2 |
| IBTKα-ΔuORF3 | Initiation codon ATG to AGG of uORF3 |
| IBTKα-ΔuORF4 | Initiation codon ATG to AGG of uORF4 |
| IBTKα-ΔuORF3+4 | Initiation codon ATG to AGG of uORF3 and uORF4 |
| **Reporters in Figure 3B-derived from IBTKα-uORF2-Luc** | |
| IBTKα-ΔuORF1-uORF2-Luc | Initiation codon ATG to AGG of uORF1 in IBTKα-uORF2-Luc |
| **Reporters in Figure 4A-derived from IBTKα-CDS-Luc** | |
| IBTKα-ΔSL 232-260 | Deletion of RNA stem loop sequence AGTGTGGAGGGGGAGTTCCCCTCCTCACT |
| IBTKα-Heterologous SL | Substitution of AGTGTGGAGGGGGAGTTCCCCTCCTCACT with CTGCAGTGGTGGAGCTTCCACCACTGCAG |
| **Reporters in Figure 4B-derived from IBTKα-CDS-Luc** | |
| IBTKα-ΔuORF1+2+3+4 | Initiation codon ATG to AGG of uORF1, uORF2, uORF3, and uORF4 |
| IBTKα-ΔuORF1+2+3+4 + ΔSL | Initiation codon ATG to AGG of uORF1, uORF2, uORF3, and uORF4 along with deletion of SL sequence AGTGTGGAGGGGGAGTTCCCCTCCTCACT |
| **Reporters in Figure 5-derived from IBTKα-CDS-Luc** | |
| IBTKα-ΔuORF2+ΔSL 232-260 | Initiation codon ATG to AGG of uORF2 along with deletion of SL sequence AGTGTGGAGGGGGAGTTCCCCTCCTCACT |
| IBTKα +50nt insert | Insertion of TACTATTATTTATCTTTGATTGTATCCATATGCCAACGGAAAGACCCTCT 3’ of AAGGGTCAGAC |
| **Reporters in Figure 6A-dervied from IBTKα-ΔSL 232-260** | |
| IBTKα-GADD34 21nt+ΔSL 232-260 | Substitution of 21nt from the 3^rd^ codon of uORF2 in IBTKα-ΔSL 232-260 ATTTAAAAGGGTCAGACCCCC with GADD34 21nt surrounding uORF2 start codon CCCCCGGGGTGACGTGCAGCC |
| IBTKα-ΔuORF2+GADD34 21nt+ΔSL 232-260 | Initiation codon ATG to AGG of uORF2 in IBTKα-GADD34 21nt+ΔSL 232-260 |
| **Reporters in Figure 6B-minimal IBTKα reporter** | |
| IBTKα-uORF2+SL | Minimal IBTKα-Luc reporter featuring only uORF2 and SL upstream of the Luc CDS |
| IBTKα-ΔSL | Deletion of RNA stem loop sequence in minimal IBTKα-uORF2+SL AGTGTGGAGGGGGAGTTCCCCTCCTCACT |
| IBTKα-ΔuORF2 | Initiation codon ATG to AGG of uORF2 in minimal IBTKα-uORF2+SL |
| IBTKα-GADD34 21nt+ΔSL | Substitution of 21nt from 3^rd^ codon of uORF2 in minimal IBTKα-ΔSL ATTTAAAAGGGTCAGACCCCC with GADD34 21nt surrounding uORF2 start codon CCCCCGGGGTGACGTGCAGCC |
| IBTKα-ΔuORF2+GADD34 21nt+ΔSL | Initiation codon ATG to AGG of uORF2 in minimal IBTKα-GADD34 21nt+ΔSL |
| **Reporters in Figure 6B** | |
| ATF4-CDS-Luc | 5’ leader of Mouse ATF4 inserted between CMV promoter and firefly luciferase CDS with initiation codon of uORF2 mutated to AGG |
| ATF4-IBTKα SL | Substitution of ATF4 CCTGCGGCAGCGTTGGCCTTTGCAGCGGCGGCAGC with IBTKα SL region GACAGTGTGGAGGGGGAGTTCCCCTCCTCACTCCC |
| ATF4-ΔuORF1+IBTKα SL | Initiation codon ATG to AGG of uORF1 in ATF4-IBTKα SL |

**Supplemental Figure 1:** IBTK ΔSL HEK293 cells were treated with thapsigargin (Tg) or vehicle for 6 hours and cell lysates were analyzed by polysome profiling. RNA was isolated from the polysome fractions of ΔSL cells and the level of *ATF4* transcript was measured from three biological replicates of ΔSL cells by RT-qPCR as described in the Experimental Procedures. The bar graphs show the percentage of total *ATF4* mRNA in each fraction and their shift towards fractions 4-6 during ER stress. By comparison, the *ATF4* and *GADD34* transcripts shifted toward fractions 4-6 by 6% and 11%, respectively in response to ER stress (derived from data presented in Fig. 1). There was trend for *ATF4* and *GADD34* mRNAs towards heavy polysomes with ER stress (n=3), but these shifts did not reach statistical significance. It is also noted that human *ATF4* and *GADD34* mRNAs have coding sequences 351 codons and 674 codons in length, respectively. *IBTKα* mRNA encodes a larger CDS (1353 codons) and consequently translated *IBTKα* transcripts are more abundant in the heavy fraction 6 of the sucrose gradients.


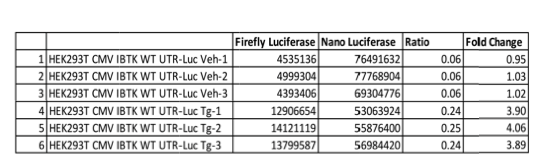


**Supplemental Figure 2:** The raw values of firefly luciferase and nano luciferase from HEK293T cells transfected with the WT version of P_CMV_-IBTKα-Luc reporter and treated with thapsigargin (Tg) or vehicle for 6 hours are given in the table. The reduction in the nano luciferase activity is observed upon ER stress.

**Supplemental Figure 3:** HEK293T cells were transfected with WT versions of P_CMV_-IBTKα-Luc reporter and were treated with thapsigargin (Tg), combination of Tg and PERK inhibitor GSK2656157, GSK2656157 by itself or vehicle. Luciferase activity (right panel) and the corresponding luciferase mRNA (left panel) were measured and presented in the bar graphs that are normalized to the non-stressed cells. Three biological replicates are depicted in the bar graphs. The * symbol is equal to p-value ≤ 0.05, ** is equal to p-value $\leq0.01,$# is equal to p-value ≤ 0.0001.

**Supplemental Figure 4:** HEK293T cells were transfected with WT and the indicated mutant versions of P_CMV_-IBTKα-Luc reporters and were treated with thapsigargin (Tg) or vehicle. Mutant versions of P_CMV_-IBTKα-Luc reporter included an insertion of a 50nt or a 100nt segment that extended the distance between the uORF2 stop codon and the SL. Luciferase activity were measured and presented in the bar graphs that are normalized to the WT IBTKα-Luc activity in non-stressed cells. Three biological replicates are depicted in the bar graphs, with values in parentheses indicating the ER stress induction for each group calculated by taking the ratio of Luc activity of Tg treated samples compared to vehicle treated. The # symbol is equal to p-value ≤ 0.0001 and ns represents non-significant.

**Supplemental Figure 5**: HEK293 WT and IBTKα KO cells were treated with thapsigargin (Tg) or vehicle for 6, 8 and 10 hours and the levels of IBTKα, p-eIF2α, total eIF2α, ATF4, CHOP, GADD34 and actin proteins were measured by immunoblot analyses. Protein lysate collected from HEK293T expressing FLAG tagged IBTKα was also loaded in the gel used for probing IBTKα. The molecular weight markers are shown in kDa.
